# Early Markers of Glycaemic Control in Children with Type 1 Diabetes Mellitus

**DOI:** 10.1371/journal.pone.0025251

**Published:** 2011-09-26

**Authors:** Samuel W. Cutfield, José G. B. Derraik, Peter W. Reed, Paul L. Hofman, Craig Jefferies, Wayne S. Cutfield

**Affiliations:** 1 Liggins Institute, University of Auckland, Auckland, New Zealand; 2 Starship Children's Hospital, Auckland District Health Board, Auckland, New Zealand; 3 National Research Centre for Growth and Development, University of Auckland, Auckland, New Zealand; Pennington Biomedical Research Center, United States of America

## Abstract

**Background:**

Type 1 diabetes mellitus (T1DM) may lead to severe long-term health consequences. In a longitudinal study, we aimed to identify factors present at diagnosis and 6 months later that were associated with glycosylated haemoglobin (HbA_1c_) levels at 24 months after T1DM diagnosis, so that diabetic children at risk of poor glycaemic control may be identified.

**Methods:**

229 children <15 years of age diagnosed with T1DM in the Auckland region were studied. Data collected at diagnosis were: age, sex, weight, height, ethnicity, family living arrangement, socio-economic status (SES), T1DM antibody titre, venous pH and bicarbonate. At 6 and 24 months after diagnosis we collected data on weight, height, HbA_1c_ level, and insulin dose.

**Results:**

Factors at diagnosis that were associated with higher HbA_1c_ levels at 6 months: female sex (p<0.05), lower SES (p<0.01), non-European ethnicity (p<0.01) and younger age (p<0.05). At 24 months, higher HbA_1c_ was associated with lower SES (p<0.001), Pacific Island ethnicity (p<0.001), not living with both biological parents (p<0.05), and greater BMI SDS (p<0.05). A regression equation to predict HbA_1c_ at 24 months was consequently developed.

**Conclusions:**

Deterioration in glycaemic control shortly after diagnosis in diabetic children is particularly marked in Pacific Island children and in those not living with both biological parents. Clinicians need to be aware of factors associated with poor glycaemic control beyond the remission phase, so that more effective measures can be implemented shortly after diagnosis to prevent deterioration in diabetes control.

## Introduction

Type 1 diabetes mellitus (T1DM) may lead to severe long-term health consequences, such as renal failure, blindness, as well as heart and cerebrovascular disease [1], [2]. Previous data have shown that intensive therapy to maintain blood glucose concentrations near the normal range delays the onset and slows the progression of adverse outcomes associated with T1DM, such as retinopathy, nephropathy and neuropathy [2]. Glycosylated haemoglobin (HbA_1c_) monitoring has been shown to improve glycaemic control when added to conventional blood glucose testing, and it is now standard practice in the management of patients with T1DM. Thus, the monitoring of HbA_1c_ levels may minimize possible short- and long-term adverse health outcomes. For example, therapy to lower HbA_1c_ from 7.9 to 7.0% reduced the risk of microvascular complications by 25% [3].

To minimize the likelihood of long-term complications, it would be valuable to identify early on those groups where diabetes control is likely to be poor (arbitrarily defined as HbA_1c_ >9%) [4].

By using predictors of metabolic control based on data collected at diagnosis or shortly after, closer monitoring of at-risk subjects would be possible. Early intervention and monitoring in these subjects could potentially reduce the likelihood of severe negative health outcomes associated with sustained hyperglycaemia.

HbA_1c_ remains the simplest indicative measure of diabetes control. Several studies have consequently examined a number of physiological and environmental factors that may affect HbA_1c_ levels in diabetic patients, such as ethnicity [5]–[10], socio-economic status (SES) [6], [11], [12] and family living arrangements [10], [13], [14]. However, these cross-sectional studies looked at correlations between specific factors and HbA_1c_, and not their ability to predict HbA_1c_ in the long-term. Further, the remission or ‘honeymoon’ phase occurs in most children shortly after T1DM diagnosis [15]–[18]. This remission phase is characterized by temporary recovery of residual β-cell function that typically lasts up to 6 months [19], and it is usually defined as requiring an insulin dose <0.5 U/kg/day [15], [17], [18]. During this phase there is a reduction in insulin dose and good glycaemic control is readily achievable [20]. As a result, during the remission phase diabetic children may have risk factors for later poor glycaemic control that are not initially evident. Thus, our retrospective analysis of longitudinal data aimed to identify factors present at diagnosis and 6 months later that are associated with HbA_1c_ levels at 24 months after T1DM diagnosis, so that diabetic children at risk of poor glycaemic control in particular, may be identified.

## Methods

### Subjects

The Endocrinology Service at Starship Children's Health (Auckland, New Zealand) provides specialist care for all children <15 years of age (yr) diagnosed with T1DM in the Auckland region, with case ascertainment levels of over 95% [21]. These children are reviewed by this service at least once every three months. New Zealand has a social security system that provides medical care free of charge, so that direct costs of T1DM to patients' families are minimal and income is not a direct impediment for take-up of medical care.

Demographic and clinical data were collected from the Starship Children's Hospital Diabetes Database (Starbase), with additional information obtained from hospital records as required. The database includes all children diagnosed with T1DM in the Auckland region, drawing from a total population of approximately 1.3 million [22].

For this study, data on all children <15 yr diagnosed with T1DM between 1 January 2000 and 31 December 2008 were collected. Subjects were included in the study if the following criteria were met: blood tests showed T1DM antibodies glutamic acid decarboxylase (GAD) and/or islet antigen 2 (IA2) at presentation, ongoing requirement for insulin, and regular attendance (≥3 visits per annum) to the Paediatric Diabetes Service at Starship. Subjects who were antibody negative were excluded to remove those with maturity onset diabetes of the young (MODY) or T2DM. Subjects were also excluded if they had been on an insulin pump within 2 years of diagnosis, had data at 6 or 24 months missing, had syndromes associated with impaired cognitive ability, had coeliac disease, or were receiving drugs known to influence insulin sensitivity. Ethics approval to conduct this study was granted by the Auckland District Health Board Research Review Committee (study number ADHB 5013).

### Study Parameters

Data on demography, auxology and biochemistry were collected at presentation, at 6±2 months and 24±3 months after diagnosis. Parameters collected at diagnosis were: age, sex, weight, height, ethnicity, family living arrangement, SES, T1DM antibody titre, venous pH and bicarbonate. At 6 and 24 months after presentation at clinic, we collected data on weight, height, HbA_1c_ level, and insulin dose.

Ethnicity was recorded by self-report using a priority system, such that if multiple ethnicities were selected, the patient was assigned to a single ethnicity, following a hierarchical classification of Maori, Pacific Islander, Other, and then European [23]. Those participants described as “Other” were of ethnicities not otherwise stated (e.g. Indian, South-East Asian, African and Middle Eastern). Family living arrangement was defined as either living with both biological parents in the same household, or as living under alternative arrangements such as the care of a single-parent, blended family, foster parents, grandparents, etc.

The New Zealand Deprivation Index 2006 (NZDep2006) [24] was used as the indicator of SES [6]. This index uses household census data reflecting nine aspects of material and social deprivation, giving scores to New Zealand residential addresses from 1–10, where 1 represents the most affluent and 10 the most deprived [25]. Scores are derived from units covering a relatively small area, each reflecting approximately 90 people [24]. These units correspond to “domicile codes” recorded in the New Zealand National Health Index database [26], so that NZDep2006 scores can be assigned to individual patients based on residential address.

Height was measured to the nearest 0.1 cm using a wall-mounted Harpenden stadiometer (Holtain, Crosswell, UK). Weight was measured to the nearest 0.1 kg, with the participant in light clothing, by electronic scales. Body mass index standard deviation scores (BMI SDS) were calculated using The University of Manchester child obesity calculator based on British 1990 growth reference data [27]. HbA_1c_ was measured with a DCA 2000 Analyzer (Bayer, Elkhart, IN, USA), which was standardised to laboratory-tested HbA_1c_ on a monthly basis. DKA occurrence was assessed by blood gases using pH and HCO3-. DKA severity was categorized as mild (venous pH<7.3 or bicarbonate <15 mmol/l), moderate (pH<7.2, bicarbonate <10 mmol/l) or severe (pH<7.1, bicarbonate <5 mmol/l) [28]. Autoantibodies to GAD and IA2 were measured using RSR ELISA kits (RSR Ltd, Cardiff, UK).

### Data Analysis

Data were analysed using JMP 8 (SAS Inc. NC, USA). Univariate comparisons were investigated with parametric t-tests or linear regression, as appropriate. Non-parametric tests of the same data made no material difference to the results and are not presented. Multivariate analyses were undertaken using the standard least squares approach. Individual variables associated in univariate tests were combined in a step-wise fashion to identify the multivariate model with the greatest r^2^, where the evidence for each variable was p<0.05. Statistical significance was defined as p<0.05.

Data are presented as mean ± SEM, unless otherwise stated.

## Results

### Diagnosis

A total of 450 children diagnosed with T1DM were recorded in Starbase between 2000 and 2008, from which 229 met all the inclusion criteria and were consequently incorporated into this study. Subjects were excluded due to: incomplete data, moving to another region, or diagnosis outside Auckland (162); insulin pump therapy within the first 2 years of the study period (19); coeliac disease (20); absence of T1DM antibodies (15); and syndromes affecting intellectual capacity (5).

There were no differences in any of the demographic characteristics between the total group and the subjects recruited into this study, which are summarised in Table 1. The majority of children were from European families of average affluence, although overall Europeans also had better SES than all other groups (Table 1). Pacific Islanders were considerably heavier than subjects of other ethnicities (p<0.01; Table 1). This trend remained throughout the study period, so that 67% of Pacific Island children were obese (BMI SDS greater than the 95^th^ percentile) throughout the study. Pacific Islanders were of lower SES than all groups but Maori (Table 1). The majority of children (86%) were living with both biological parents (Table 1). Females were more likely than males to be in DKA at diagnosis (30 vs 19%, p<0.05), and females in DKA at diagnosis were 4-fold more likely to be in severe DKA than males (36 vs 9.0%, p<0.05).

**Table 1:** Demographic characteristics of subjects at diagnosis.

Factor		N (%)	Age (years)	Males (%)	BMI SDS	NZDep2006	DKA at D_x_ (%)
Sex	Female	111 (49%)	8.3±0.3	-	0.56±0.10***	4.91±0.27	30*
	Male	118 (52%)	8.4±0.3	-	1.04±0.10	4.98±0.27	19
Family living arrangement	Both biological parents	196 (86%)	8.0±0.3	50	0.82±0.07	4.72±0.20**	22
	Other arrangements	33 (14%)	7.5±0.6	61	0.83±0.19	6.24±0.54	33
Ethnicity	European	157 (69%)	8.6±0.3	53	0.63±0.07^a^	4.22±0.21^a^	20
	Maori	16 (7%)	6.7±0.8	50	0.97±0.24^a^	6.44±0.77^bc^	31
	Other	32 (14%)	8.9±0.6	34	0.76±0.18^a^	5.59±0.53^c^	28
	Pacific Islander	24 (11%)	7.7±0.7	63	1.99±0.22^b^	7.79±0.43^b^	27

Where applicable, data are mean ± SEM.

*p<0.05,

**p<0.01, and

***p<0.001 for comparisons between groups. Different letters indicate significant differences (p<0.05) between groups.

### 6 months after diagnosis

Several demographic factors at diagnosis were associated with worse glycemic control as reflected in higher HbA_1c_ levels at 6 months (HbA_1c 6mo_): female sex (p<0.05), lower SES (p<0.01), non-European ethnicity (p<0.01), younger age (p<0.05), and having a first degree relative with T1DM (p<0.05). The remission or ‘honeymoon’ phase (insulin dose <0.5 U/kg/day) was present in 33% of children, and it was strongly associated with ethnicity (17% of Pacific Island vs 37% of European children; p<0.001). Children within the remission phase had a mean HbA_1c_ of 7.1%, and these were predominantly European (75%). Notably, the HbA_1c_ was higher among Pacific Island children in the remission phase (7.5%). Neither the presence nor severity of DKA at diagnosis influenced HbA_1c 6mo_.

### 24 months after diagnosis

**Figure 1:**
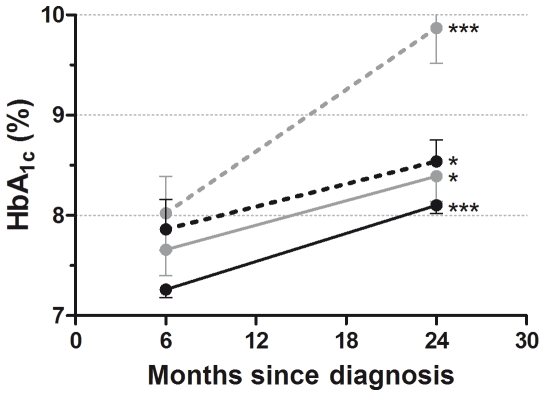
Mean changes in HbA_1c_ between 6 and 24 months after diagnosis for Europeans (n = 157; solid black line), Maori (n = 16; dashed black), Pacific Islanders (n = 24; dashed gray), and Other ethnicities (n = 32; solid gray). Data are mean ± SEM. *p<0.05 and ***p<0.001 for temporal changes within each ethnic group.

Although HbA_1c 6mo_ was associated with HbA_1c 24mo_ (p<0.001), it had a weak positive predictive value (r^2^ = 0.16). However, demographic and clinical characteristics obtained at diagnosis were associated with higher HbA_1c 24mo_: lower SES (p<0.001), Pacific Island ethnicity (p<0.001), not living with both biological parents (p<0.05), and greater BMI SDS (p<0.05). The effect of ethnicity on HbA_1c_ levels at 6 and 24 months following diagnosis was again marked. Pacific Island children, who had worse glycaemic control at 6 months, displayed HbA_1c_ levels that had deteriorated more markedly than other groups by 24 months (Fig. 1), so that mean HbA_1c_ in Pacific Islanders were 9.9±0.4% compared to 8.1±0.1% (p<0.001) in European children. HbA_1c_ levels were also higher in children with living arrangements other than living with both biological parents (8.9±0.3 vs 8.3±0.1%, p<0.05). At 24 months after diagnosis, there were only three children (1.3%), of whom two were European, still in the remission phase.

### Predictive equation

A multivariate regression equation was developed to identify demographic and clinical factors 6 months after diagnosis that predicted glycaemic control at 24 months after diagnosis (r^2^ = 0.33, p<0.001):
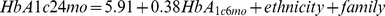



Where, for *ethnicity*, Pacific Island  =  +1.06, Other  =  -0.27, European  =  -0.37, and Maori  =  -0.42 for *family*, living with both biological parents  =  -0.28, and other living arrangements  =  +0.28.

The model was not strengthened with the addition of SES, adiposity, or insulin dose at 6 months after diagnosis.

Pacific Island children were markedly heavier than all other ethnicities, but particularly Europeans (BMI SDS of 1.98±0.21 vs 0.64±0.07 at 6 months, and 2.02±0.16 versus 0.75±0.07 at 24 months). However, in the multivariate analysis it was ethnicity and not BMI SDS that influenced HbA_1c_ levels at 6 and 24 months, suggesting that ethnicity itself rather than body composition might have affected glycaemic control across the entire study group. The effect of SES was also lost in multivariate analysis, suggesting that ethnicity rather than SES was the major influence on HbA_1c_ levels.

## Discussion

In a regional cohort of children with T1DM, we found that Pacific Islanders and those of low SES had higher HbA_1c_ levels at 6 months from diagnosis, with an even greater deterioration in glycaemic control in Pacific Island children after 24 months. In addition, children not living with both biological parents also displayed poorer glycaemic control.

Cross-sectional analyses have previously shown that both ethnicity and to a lesser degree SES influence HbA_1c_ levels in patients with diabetes [5], [6], [10], [11]. Other studies have observed that children and adolescents from ethnic minorities display worse glycaemic control [6], [10], [29]–[31]. African American children and adolescents with T1DM showed a similar marked difference in HbA_1c_ values, which were 1.3 to 1.5% higher than European Americans [5], [10]. Less dramatic differences have been seen in other ethnicities such as New Zealand Maori (HbA_1c_ 1.0% higher than Europeans) [6] and American Hispanic children (HbA_1c_ 0.5% higher) [11]. However, even though ethnicity is linked to diabetes control (HbA_1c_), the key characteristics of minority groups associated with poor glycaemic control have not been well-described. Nonetheless, ethnicity and SES are often associated, and minority ethnic groups tend to be more socially disadvantaged, experiencing higher poverty rates and worse health status [32], [33]. Although SES can be associated with poor glycaemic control in children, this is not always the case [5], [6], [34], [35]. In this study, we observed that SES on its own influenced HbA_1c_, but when combined with ethnicity in a multivariate regression, ethnicity was the greater influence and there was no independent SES effect.

Family living arrangement was also an important factor determining diabetes management, and children living with both their biological parents were associated with better glycaemic control 24 months after diagnosis. Other studies have shown that living with a single parent is associated with poor glycaemic control in children and adolescents [5], [10], [36]–[39]. In our study, single-parents were combined with other living arrangements, mainly those living in blended-parent families, for which similar association with poor glycaemic control has been previously observed [40].

With awareness of the risk factors associated with poor glycaemic control, physicians can assist those patients most at risk of inadequate diabetes management in the long-term. This study has identified a number of primary risk factors: Pacific Island ethnicity, lower SES, higher HbA_1c 6mo_, increased BMI SDS, and living arrangements without both biological parents. Although predictably higher HbA_1c 6mo_ was associated with higher HbA_1c 24mo_, the relationship was weak.

Recently, a study found that HbA_1c_ at 1 year in children with T1DM would not allow practitioners to predict the trajectory of glycaemic control over the subsequent 3 years [41], but other physiological and environmental factors were not included in their analysis. Thus, to better predict glycaemic control at 24 months from diagnosis (i.e. HbA_1c 24mo_), we have developed a multivariate regression equation using demographic characteristics at diagnosis and clinical features 6 months afterwards (including HbA_1c 6mo_). The equation not only identifies primary risk factors associated with inadequate diabetes management, but also enables clinicians to estimate long-term glycaemic control. The regression equation estimating HbA_1c_ 24 months after diagnosis included three factors: HbA_1c 6mo_, ethnicity, and family living arrangements. So, for example, the model predicts with 95% confidence that a Pacific Island child with an HbA_1c 6mo_ of 7.9% from a single-parent family would have HbA_1c_ 24 months after diagnosis of 10.3±0.6 (mean ± SD). Alternatively, a European child with HbA_1c 6mo_ of 7.3% living with two biological parents would have HbA_1c_ 24 months after diagnosis of 8.1±0.2.

Shortly after diagnosis, children with T1DM have a temporary but appreciable residual insulin secretion (remission phase), which may disguise attributes associated with poor glycaemic control. We observed that 6 months after diagnosis a third of T1DM children were in the remission phase, but by 24 months virtually no children remained in this phase. These data are consistent with values obtained in other studies [15], [16], [42], [43].

In the months after diagnosis clinicians may not adjust for the impact of the remission phase. In this period glycemic control can appear satisfactory, but it is in fact too high for the remission phase. Other studies have not examined changes in HbA_1c_ levels from within and then beyond this remission phase. Thus, in the first few months following diagnosis, most children with T1DM appear to have good glycaemic control, creating an illusion that their diabetes is well managed. Once the remission phase is over, HbA_1c_ levels are more likely to reflect actual diabetes management, rather than endogenous insulin secretion.

The end of this remission phase unmasks non-compliant patients, which show a dramatic increase in HbA_1c_ levels, signalling poor glycaemic control. In our study, although Pacific Island children initially had only a slightly higher HbA_1c 6mo_ than European children, this difference was considerably amplified after the remission phase. As a result, there was a marked deterioration in glycaemic control 24 months after diagnosis among Pacific Island children, whose mean HbA_1c 24mo_ was 9.9% compared to 8.1% among Europeans. Importantly, the remission phase can be prolonged with more intensive insulin treatment and could be of considerable benefit to Pacific Island children [44], [45]. Interestingly, the remission phase among Pacific Island children was very short, and 83% of subjects transitioned out of this phase within 6 months of diagnosis which occurred more than twice as quickly as for European children. Thus, inadequate diabetes management characteristics were likely to have been present among Pacific Island children shortly after diagnosis.

Obesity among Pacific Islanders is extremely high, and a recent representative study of 1011 Pacific Island adults in Auckland showed that 74% of women and 53% of men were obese [46]. Obesity among Pacific Island children is also very common (approximately 24–26%) and rates are considerably higher than in other ethnic groups [47], [48]. The rate of obesity in diabetic Pacific Island children in this study was even higher (67%), and we speculate that it may be a major contributor to their poor glycaemic control and shorter remission phase. However, obesity in European children was found to be only weakly associated with HbA_1c._

Pacific Islanders suffer from greater morbidity and mortality than other ethnic groups in New Zealand [49], with greater prevalence of diabetes and metabolic syndrome than Europeans [50]. A study of over 13,000 patients with T1DM and T2DM attending general practices in the South Island showed that Pacific Islanders/Maori also had poorer glycaemic control than Europeans [51]. Although genetics may be a factor [52], cultural and environmental factors other than SES are likely to be at play as observed elsewhere [33], [53], [54], and Pacific Islanders in this study are likely to be representative of minority ethnicities in other countries.

We also found that the prevalence and severity of DKA at diagnosis was considerably greater among girls. Further, girls had worse glycaemic control than boys, a pattern that has been previously observed [29]. Differences in metabolism and/or pubertal status between the sexes may account for these differences, and Hoffman *et al*. showed that early pubertal girls were less insulin sensitive than boys, but that this difference was compensated by increased insulin secretion [55].

### Conclusions

Deterioration in glycaemic control typically occurs from 6 months to 24 months after diagnosis in children with T1DM. This pattern is particularly marked in Pacific Island children and in those not living with both biological parents. Clinicians need to be aware of factors associated with poor glycaemic control beyond the remission phase, so that more effective measures can be implemented shortly after diagnosis to prevent deterioration in diabetes management.
